# Identification of Small-Molecule Inhibitors Targeting Porphyromonas gingivalis Interspecies Adherence and Determination of Their *In Vitro* and *In Vivo* Efficacies

**DOI:** 10.1128/AAC.00884-20

**Published:** 2020-10-20

**Authors:** Mohammad Roky, Jinlian Tan, Maryta N. Sztukowska, John O. Trent, Donald R. Demuth

**Affiliations:** a Department of Oral Immunology and Infectious Diseases, University of Louisville School of Dentistry, Louisville, Kentucky, USA; b Department of Microbiology and Immunology, University of Louisville School of Medicine, Louisville, Kentucky, USA; c Department of Medicine, University of Louisville School of Medicine, Louisville, Kentucky, USA; d University of Information Technology and Management, Rzeszow, Poland

**Keywords:** virtual screening, drug discovery, biofilms, *Porphyromonas gingivalis*

## Abstract

Porphyromonas gingivalis is one of the primary causative agents of periodontal disease and initially colonizes the oral cavity by adhering to commensal streptococci. Adherence requires the interaction of a minor fimbrial protein (Mfa1) of P. gingivalis with streptococcal antigen I/II (AgI/II). Our previous work identified an AgI/II peptide that potently inhibited adherence and significantly reduced P. gingivalis virulence *in vivo*, suggesting that this interaction represents a potential target for drug discovery. To develop targeted small-molecule inhibitors of this protein-protein interaction, we performed a virtual screen of the ZINC databases to identify compounds that exhibit structural similarity with the two functional motifs (NITVK and VQDLL) of the AgI/II peptide. Thirty three compounds were tested for *in vitro* inhibition of P. gingivalis adherence and the three most potent compounds, namely, N7, N17, and V8, were selected for further analysis. The *in vivo* efficacy of these compounds was evaluated in a murine model of periodontitis. Treatment of mice with each of the compounds significantly reduced maxillary alveolar bone resorption in infected animals. Finally, a series of cytotoxicity tests were performed against human and murine cell lines. Compounds N17 and V8 exhibited no significant cytotoxic activity toward any of the cell lines, whereas compound N7 was cytotoxic at the highest concentrations that were tested (20 and 40 μM). These results identify compounds N17 and V8 as potential lead compounds that will facilitate the design of more potent therapeutic agents that may function to limit or prevent P. gingivalis colonization of the oral cavity.

## Abstract (precis)

Porphyromonas gingivalis is one of the primary causative agents of periodontal disease and initially colonizes the oral cavity by adhering to commensal streptococci. Adherence requires the interaction of a minor fimbrial protein (Mfa1) of P. gingivalis with streptococcal antigen I/II (AgI/II). Our previous work identified an AgI/II peptide that potently inhibited adherence and significantly reduced P. gingivalis virulence *in vivo*, suggesting that this interaction represents a potential target for drug discovery.

## INTRODUCTION

The oral microbiome comprises a complex and diverse community of microorganisms containing hundreds of species of bacteria, as well as fungi, viruses, archaea, and protozoa (1). Disruption of normal host-biofilm homeostasis can induce significant changes in the composition of this microbial community and lead to the development of periodontal disease, one of the most common and widespread human diseases in the world (2). Periodontal disease is characterized by polymicrobial-mediated chronic inflammation that is induced by this dysbiotic microbial community which leads to the destruction of the tissues supporting the teeth, alveolar bone resorption, and ultimately tooth loss (3) Periodontal disease is also associated with other systemic diseases, such as cardiovascular disease, rheumatoid arthritis, diabetes, Alzheimer’s disease, and pregnancy complications. (4–6). Treatment of periodontal disease typically involves the mechanical removal of dental plaque by scaling and root planing, antibiotic therapy, and if necessary gingival surgery to reduce the depth of the subgingival pocket (7).

Although periodontal disease is considered to be a polymicrobial infection, Porphyromonas gingivalis has been suggested to function as a keystone pathogen that can alter host innate immune functions, leading to dysbiosis and chronic inflammation (3, 8, 9). The primary niche of P. gingivalis is the subgingival pocket (10, 11), but initial colonization of the oral cavity by P. gingivalis occurs in the supragingival biofilm where the organism can adhere to primary colonizing organisms, such as oral streptococci (12, 13). Adhesion of P. gingivalis to streptococci is primarily driven by a protein-protein interaction between the minor fimbrial antigen (Mfa1) of P. gingivalis and specific members of the streptococcal surface antigen I/II family of proteins (e.g., SspB of Streptococcus gordonii) (14–16). As one of the initial interactions contributing to P. gingivalis colonization, this protein-protein interaction represents an ideal candidate for therapeutic intervention.

The interaction of Mfa1 with SspB has been well characterized. Deap et al. identified two discrete motifs of SspB comprised of the amino acids NITVK and VQDLL and showed that these motifs are essential for the interaction with Mfa1 (17). Daep et al. also showed that these motifs in SspB closely resembled the functional motifs of the eukaryotic nuclear receptor (NR) box protein-protein interaction domain (17, 18). Furthermore, a synthetic peptide containing both NITVK and VQDLL (designated BAR) potently inhibited P. gingivalis adhesion to S. gordonii and significantly reduced P. gingivalis virulence in a murine model of periodontitis (16, 18). Subsequently, Patil et al. synthesized a series of highly active small-molecule peptidomimetics of BAR using a click chemistry approach and demonstrated that these compounds exhibited no toxicity toward a variety of human cells and cell lines (19, 20).

The advent of computer-assisted molecular modeling technologies and structure-based virtual screening methods provide an additional platform for rational drug design for identifying targeted small-molecule inhibitors of biologic interactions. For example, Stone et al. utilized a high-throughput virtual screening approach of the ZINC (ZINC is not commercial) database of commercially available chemical compounds to identify small-molecule inhibitors of P. gingivalis *m*-diaminopimelate dehydrogenase, an essential enzyme involved in protein and cell wall synthesis (21). In this study, we performed virtual screening of the ZINC drug-like chemical libraries to identify small-molecule homologs similar to the NITVK and VQDLL motifs of SspB. The three most potent compounds that were identified inhibited P. gingivalis adherence to streptococci and reduced P. gingivalis virulence *in vivo*. Two of these active compounds showed no significant cytotoxic activity toward a variety of human and murine cell lines and represent potential lead compounds for the development of novel therapies to limit P. gingivalis colonization of the oral cavity.

## RESULTS

### *In vitro* functional assessment of the identified ZINC library candidates.

**FIG 1:**
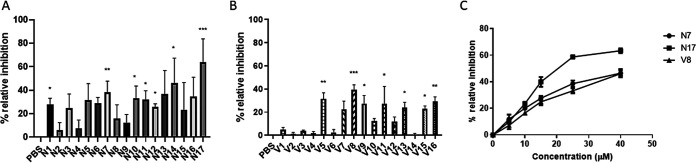
Compounds N1 to N17 (A) and V1 to V16 (B) were screened for inhibition of P. gingivalis adherence to S. gordonii at an initial concentration of 40 μM. Relative inhibition was normalized to the PBS control. Data were analyzed by one-way ANOVA followed by Dunnett’s multiple-comparison test using PBS as the control. Significance was defined as follows: *, *P* < 0.05; **, *P* < 0.01; ***, *P* < 0.001. (C) Dose-dependent inhibition of P. gingivalis adherence by N7, N17, and V8. Compounds were tested at concentrations of 5, 10, 15, 25, and 40 μM, and inhibition was normalized to the PBS control.

After virtual screening, a total of 33 commercially available molecules with structural similarity to either the NITVK or VQDLL motifs were identified and obtained for functional testing. Using the two species biofilm model described by Patil et al. (19), the *in vitro* effectiveness of the compounds was determined by their ability to inhibit P. gingivalis adherence to S. gordonii and subsequent biofilm formation at an initial concentration of 40 μM. As shown in Fig. 1A, 7 of the 17 compounds selected based on similarity with the NITVK motif exhibited significant inhibition of adherence and biofilm formation. The two most potent inhibitors were selected for further analysis, namely, N7 (40% inhibition) and N17 (60% inhibition). In addition, 7 of the 16 selected compounds with similarity with the VQDLL motif exhibited significant inhibition (Fig. 1B). The most potent of these, compound V8, inhibited biofilm formation by ≥40% and was selected for further study. Subsequently, dose-dependent inhibition studies were carried out for compounds N7, N17, and V8 using concentrations of 5, 10, 15, 25, and 40 μM. As shown in Fig. 1C, each of these compounds inhibited P. gingivalis adherence and biofilm formation in a dose-dependent manner. Thus, compounds that are similar to both of the functional domains of the streptococcal SspB protein inhibited P. gingivalis/S. gordonii adherence *in vitro*.

**FIG 2:**
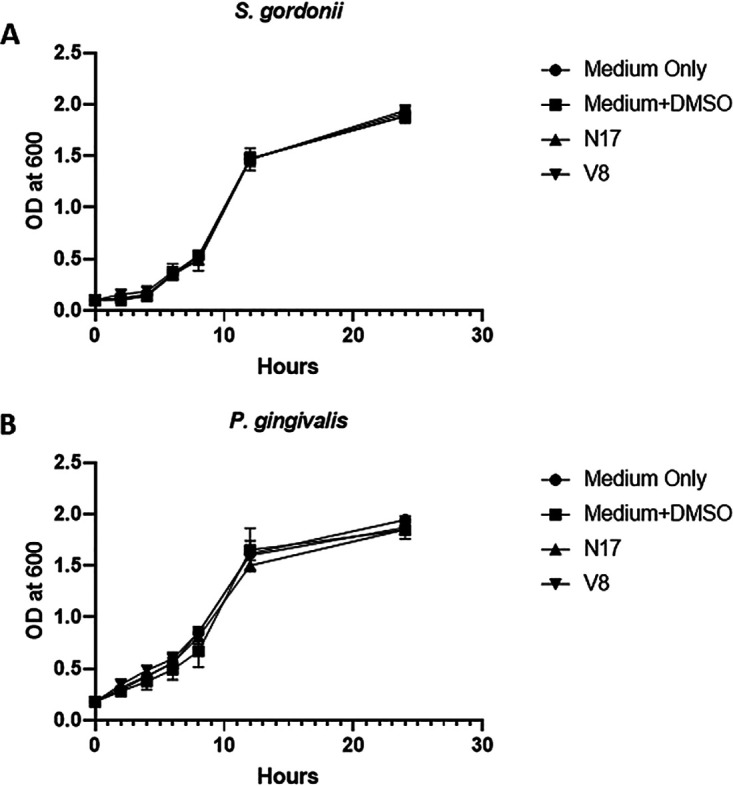
S. gordonii (A) and P. gingivalis (B) were grown for 24 h in medium containing 40 μM of each compound, and growth was quantified at various time points by measuring the OD_600_ for each culture. One-way analysis of variance (ANOVA) was performed to determine statistical significance.

**FIG 3:**
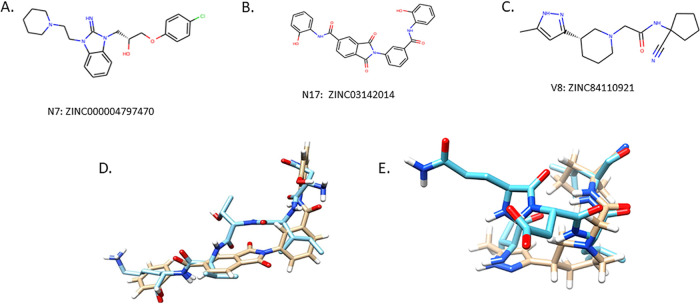
Chemical structures of N7: 1-(4-chlorophenoxy)-3-{2-imino-3-[2-(piperidin-1-yl)ethyl]-2,3-dihydro-1H-1,3-benzodiazol-1-yl}propan-2-ol hydrochloride (A), N17: N-(2-hydroxyphenyl)-2-{3-[(2-hydroxyphenyl)carbamoyl]phenyl}-1,3-dioxo-2,3-dihydro-1H-isoindole-5-carboxamide (B), and V8: N-(1-cyanocyclopentyl)-2-[3-(5-methyl-1H-pyrazol-3-yl)piperidin-1-yl] acetamide (C). (D) Overlay of compound N17 on the NITVK motif. (E) Overlay of compound V8 on the VXXLL motif. In the overlay images, compounds N17 and V8 are indicated with the tan backbones, and the AgI/II motifs are shown with the blue backbones.

To confirm that N7, N17, and V8 function by inhibiting P. gingivalis adherence to S. gordonii rather than acting as antibacterial agents, the microbicidal activity of the compounds was determined by growing the organisms in medium containing 40 μM of each compound for 24 h. As shown in Fig. 2, the growth curves obtained for S. gordonii and P. gingivalis cultures grown in the presence of N7, N17, or V8 were not significantly different from the growth curves for cultures grown in medium alone or in medium containing 0.1% dimethyl sulfoxide (DMSO). These results indicate that none of the compounds possess antibacterial activity. The structures of compounds N7, N17, and V8 are shown in Fig. 3A, B, and C, respectively. In addition, overlays of compound N17 with the NITVK motif and compound V8 with the VXXLL motif of Ag I/II are shown in Fig. 3D and E, respectively.

### Small-molecule inhibition of P. gingivalis virulence.

**FIG 4:**
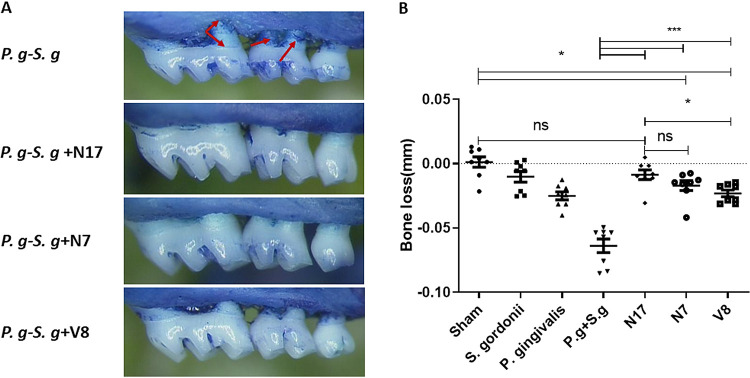
*In vivo* inhibition of P. gingivalis virulence. (A) Representative images of maxillary molars of mice infected with P. gingivalis and S. gordonii and treated with compound N7, N17, or V8 at a concentration of 40 μM. (B) Quantification of alveolar bone loss. Bone loss was determined by measuring the distance from the alveolar bone crest to the cemento-enamel junction, and values were normalized against sham-infected mice. Statistically significant differences were determined using one -way ANOVA. *, *P* < 0.05; ***, *P* < 0.001; ns, not significant.

The *in vivo* activity of compounds N7, N17, and V8 was determined using a murine model of periodontitis as described by Deap et al. (16). Infection of BALB/c/ByJ mice with P. gingivalis has previously been shown to induce key inflammatory mediators, e.g., interleukin-1 (IL-1), tumor necrosis factor (TNF), and IL-17 (22–24), leading to the resorption of alveolar bone anchoring the teeth, the primary clinical presentation of periodontitis in humans. Therefore, P. gingivalis virulence was assessed by alveolar bone loss, which was determined by measuring the distance from the alveolar bone crest (ABC) to the cemento enamel junction (CEJ). Figure 4A shows representative images of the maxillary jaws of treated and untreated animals. Infected but untreated mice exhibited an uneven ABC and more extensive exposure of tooth roots (arrows in Fig. 4A) than control animals or mice treated with N7, N17, or V8. The quantification of bone loss for each group of mice is shown in Fig. 4B. Consistent with our previous results (16), mice infected with both S. gordonii and P. gingivalis showed significantly greater alveolar bone loss than sham-infected mice or animals infected with S. gordonii or P. gingivalis alone. In contrast, infection of mice with S. gordonii and P. gingivalis in the presence of N7, N17, or V8 resulted in a significant reduction in alveolar bone loss (*P* < 0.001). Indeed, the amount of bone loss observed in animals treated with N17 was not significantly different from sham-infected animals. Mice that were treated with N7 or V8 exhibited a modest increase in bone loss relative to the sham-infected group but had significantly reduced bone loss compared with untreated mice.

### Determination of cytotoxic activity of compounds N7, N17, and V8.

**FIG 5:**
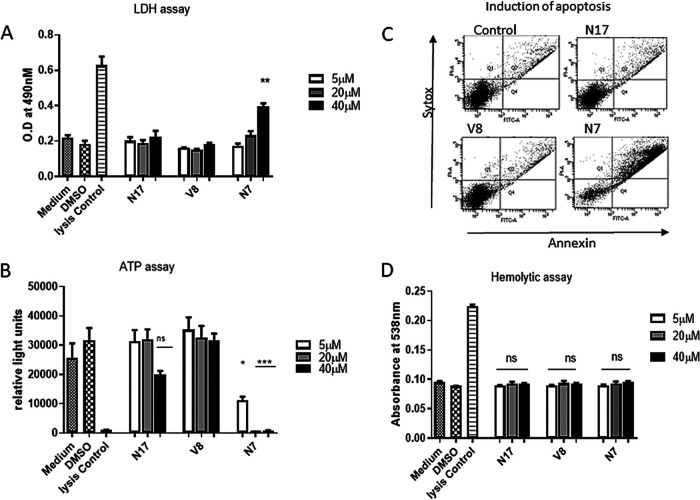
Cytotoxic activity of N7, N17, and V8 against human TIGK cells was measured by determining LDH release into the cell free supernatant (A), ATP levels in TIGK cells treated with compounds (B), and induction of TIGK cell apoptosis after treatment with compounds at concentration of 40 μM (C). The bottom left quadrant represents live cells, the bottom right quadrant represents cells exhibiting early apoptosis, and the top right quadrant represents late apoptotic cells. (D) Hemolytic activity of the compounds against human blood cells after incubation with compounds at concentrations of 5, 20, and 40 μM. Significant differences were determined by comparing experimental samples to the medium only and medium/DMSO controls. *, *P* < 0.05; ***, *P* < 0.001; ns, not significant.

To assess compound cytotoxicity toward eukaryotic cells, a series of toxicity tests were carried out using telomerase immortalized human gingival keratinocytes (TIGKs) and the murine J774.A1 and human HL-60 cell lines. These tests included measuring lactate dehydrogenase (LDH) release and overall cell viability (cellular ATP levels) of cells after treatment with each compound. In addition, the apoptotic and hemolytic activity of each compound was determined. As shown in Fig. 5A to C, respectively, LDH release, ATP, levels and apoptosis of TIGK cells treated with compounds N17 and V8 did not significantly differ from the negative controls. Quantification of the apoptosis data shown in Fig. 5C is summarized in Table 1. In contrast, exposure of TIGK cells to 40 μM N7 resulted in a significant increase in LDH release, suggesting that this compound may disrupt the integrity of the cell membrane. Exposure to N7 also significantly reduced cellular ATP levels (Fig. 5B) and significantly reduced the live cell count with a concomitant increase in the number of early and late apoptotic cells (Fig. 5C and Table 1). Finally, none of the compounds exhibited hemolytic activity toward either human (Fig. 5D) or sheep (see Fig. S1 in the supplemental material) red blood cells.

**TABLE 1:** Quantification of early and late apoptotic TIGK cells from Fig. 5C

Treatment	Concn (μM)	Live cells (%)	Early apoptosis (%)	Late apoptosis/necrosis (%)^*a*^
Medium		92.7	2.6	2.7
Medium/DMSO		91.3	1.4	3.9
Medium/H_2_O_2_		56.6	4.9	21.9*
N17	5	90.7	2.9	4.2
	20	92.4	2.4	3.6
	40	91.6	1.5	5.1
V8	5	91.6	3.1	4.2
	20	92.8	2.1	3.5
	40	92.8	2.4	3.4
N7	5	86.8	2.4	8.3
	20	74.5	1.0	14.9*
	40	48.3	5.9	40.7*

a Asterisks indicate a significant increase (*P* < 0.001) in apoptotic cells relative to the medium and medium/DMSO controls.

Similar results were obtained for the J774A.1 and HL-60 cell lines. Compounds N17 and V8 had no significant effect on LDH release (see Fig. S2 in the supplemental material), ATP levels (see Fig. S3 in the supplemental material), or cellular apoptosis (Tables S1 and S2), whereas exposure to N7 resulted in significant increases in LDH release, ATP levels, and cell apoptosis at 20 μM and 40 μM. Together, these data indicate that N17 and V8 exhibit no significant cytotoxic activity toward the eukaryotic cell lines tested. In contrast, compound N7 exhibited significant cytotoxicity and may not be biocompatible.

## DISCUSSION

P. gingivalis may contribute to the initiation and progression of periodontitis by functioning as a keystone pathogen that alters host innate immune functions and leads to dysbiosis and chronic inflammation (3, 8, 9). This raises the possibility that therapeutic approaches that are specific for P. gingivalis may be effective in controlling periodontal disease. One potential therapeutic target is the interaction of Mfa1 with streptococcal AgI/II, which may contribute to the initial colonization of the oral cavity by P. gingivalis.

Structural dissection of both AgI/II (16–18) and Mfa1 (25) previously identified specific amino acids and motifs that are required for adherence of P. gingivalis with streptococci and facilitated the development of a peptide that inhibits this interaction (16). However, P. gingivalis is a highly proteolytic organism, which would likely limit the application of this peptide for therapeutic purposes. To address this limitation, small-molecule peptidomimetics have been recently synthesized and were shown to inhibit P. gingivalis adherence (19, 20, 26). In this study, we sought to identify additional small-molecule inhibitors by virtual screening of the ZINC database (https://zinc.docking.org/) (27–29) for commercially available compounds that exhibit similarity with the NITVK and VQDLL functional motifs of the streptococcal AgI/II protein. Three lead compounds, N7, N17, and V8, were identified and shown to inhibit P. gingivalis/streptococcal adherence *in vitro* and to reduce P. gingivalis virulence *in vivo*. Of the three compounds, N17 was the most potent inhibitor of P. gingivalis adherence *in vitro* and exhibited a 50% inhibitory concentration (IC_50_) of ∼18 μM, similar to the first peptidomimetic compounds based on the BAR peptide reported by Patil et al. (19). Preliminary experiments using equimolar mixtures of N17 and V8 did not indicate significant synergistic activity. One possible explanation for this is that the binding of one compound, e.g., N17, to Mfa1 may sterically hinder the association of V8. *In vivo*, it has been previously shown that the presence of S. gordonii in the murine oral cavity promotes P. gingivalis virulence (16, 20), and consistent with this finding, infection of mice with both S. gordonii and P. gingivalis induced inflammation, leading to resorption of alveolar bone. Treatment of infected animals with each of the compounds resulted in a significant reduction in P. gingivalis-mediated inflammation and bone loss. Indeed, bone loss in mice treated with compound N17 was not statistically different from sham-infected animals.

Compounds N17 and V8 exhibited little cytotoxicity against human gingival epithelial cells or human and mouse macrophage cell lines. In contrast, N7 exhibited a significant level of cytotoxicity in each of the cell culture tests that were performed. However, mice that were treated with compound N7 during the infection process did not exhibit any overt signs of distress or toxicity. This finding could be explained by the difference in the duration of exposure to the compound in the *in vitro* versus *in vivo* experiments. Animals that were treated with N7 were only transiently exposed (∼20 min) to the compound during infection with P. gingivalis, whereas cell cultures were exposed to N7 for 18 h prior to the toxicity analyses.

One potential application that we envision for these compounds would be to prevent or reduce colonization of the redeveloping oral microbiome by P. gingivalis after a patient is treated for periodontitis. Typically, the oral microbiome reforms after treatment, and disease recurrence can often occur. We speculate that topical application of these compounds, formulated in a dental varnish or mouth rinse, may direct the redevelopment of the microbial community toward a healthy rather than pathogenic biofilm by preventing recolonization of P. gingivalis. Our observation that transient exposure of animals to the compounds significantly reduced P. gingivalis virulence provides an initial proof of concept that preventing P. gingivalis colonization of the oral microbiome may result in positive clinical outcomes. In addition, we previously showed that the BAR peptide was also capable of disrupting an established biofilm containing P. gingivalis (30), which suggests that N17 and V8 may also have utility in treating existing periodontal infections.

In summary, virtual screening of the ZINC database identified three compounds that inhibited P. gingivalis adherence to oral streptococci and represent potential targeted therapeutics against periodontal disease. Two of these compounds exhibited biocompatibility with both human and mouse cells and represent lead compounds that will provide a platform for further modification to improve potency.

## MATERIALS AND METHODS

### Bacterial strains and culture conditions.

P. gingivalis ATCC 33277 was cultured in reduced Trypticase soy broth-yeast extract (TSBY) medium comprised of 30 g/liter TSB (Difco Laboratories Inc., Detroit, MI), 5% (wt/vol) yeast extract supplemented with 5 mg/liter hemin and 1 mg/liter menadione under anaerobic conditions (10% CO_2_, 10% H_2_, and 80% N_2_) at 37°C for 48 h. Reduced TSBY medium was prepared by incubating the medium under anaerobic conditions at 37°C for 24 h prior to bacterial inoculation. Streptococcus gordonii DL1 was grown in brain heart infusion (BHI) (Difco Laboratories Inc.) broth supplemented with 5% (wt/vol) yeast extract under anaerobic conditions as described above.

### Virtual screening of small-molecule inhibitors.

The structures of the NITVK and VQDLL motifs of SspB were taken directly from the Streptococcus gordonii SspB C-terminal domain crystal structure (Protein Data Bank entry 2WZA) (31). The structure was processed using the Protein Preparation Wizard in Maestro (Schrödinger release 2018-1; Schrödinger, LLC, New York, NY). The similarity searches for the NITVK and VQDLL motifs were performed with Surflex-sim version 2.601 (32) using two approaches. The first approach was to use all atoms of the peptide structures for the NITVK and VQDLL motifs as the hypothetical ligand. The second approach was to use the side chains of residues for the NITVK motif and the side chains of residues VLL for the VQDLL motif. The screened libraries were created from the ZINC (28) drug-like library (ZINC 2014 version) containing 24,877,119 compounds and the ZINC 15 (29) drug-like library (ZINC 2016 version) containing 17,244,856 compounds. The results were ranked, and the top 500 compounds of each screen were retained. The selection of compounds was based on compound score; diversity, by eliminating compounds that were structurally similar to a higher scoring compounds; and finally, compounds that were commercially readily available for purchase. Seventeen compounds and 16 compounds were purchased (MolPort SIA, Riga, Latvia) for the NITVK and VQDLL motifs, respectively.

### P. gingivalis adherence to streptococci.

To quantify P. gingivalis adherence to S. gordonii, a two-species biofilm model was employed as previously described (19, 33). Briefly, 20 ml of an overnight culture of S. gordonii was centrifuged at 5600 × *g* (MIKRO 220R, Hettich Zentrifugen) for 10 min, and the harvested cells were washed with phosphate-buffered saline (PBS; 10 mM Na_2_HPO_4_, 18 mM KH_2_PO_4_, 1.37 mM NaCl, and 2.7 mM KCl [pH 7.2]). To label S. gordonii, the cell pellet was suspended in 1 ml of PBS containing 20 μl of 5 mg/ml of hexidium iodide (Molecular Probes; Eugene, OR) and incubated in the dark for 15 min at room temperature with gentle shaking. The labeled cells were than centrifuged at 5600 × *g* for 5 min and washed twice with PBS. Finally, the cells were suspended in PBS to a final optical density at 600 nm (OD_600_) of 0.6, and 1 ml of labeled S. gordonii cells was added to each well of a 12-well microtiter plate (Greiner Bio-one, Monroe, NC) containing a circular coverslip (Fisher Scientific, Hampton, NH). Plates were incubated at 37°C for 24 h under anaerobic conditions on a rotary shaker. On the following day, cells from a 10-ml overnight P. gingivalis culture were harvested as described above and fluorescent labeling was performed by incubating the cells in 1 ml PBS containing 20 μl of 5(6)-carboxyfluorescein *N*-hydroxysuccinimide ester (4 mg/ml) (Thermo Fisher, Waltham, MA) for 30 min in the dark at room temperature with gentle shaking. After being washed two times with PBS, the labeled cells were suspended in PBS at a final OD_600_ of 0.4. While this reaction was carried out, unattached S. gordonii cells were removed from each well by aspiration, and subsequently, 1 ml of labeled P. gingivalis cells alone or with the desired concentration of the compounds to be tested was added to each well of the microtiter plates. Plates were incubated at 37°C for 24 h under anaerobic conditions. The compounds that were tested were initially dissolved in dimethyl sulfoxide (DMSO) to generate a 1,000× stock solution and subsequently diluted in the cell suspensions to obtain the working concentration. For the control wells, DMSO without a test compound was added to a final concentration of 0.1% in order to maintain a constant DMSO concentration in all wells.

Unbound P. gingivalis cells were removed from each well by aspiration, and the coverslips were washed twice with 5 ml of PBS. Biofilms were then fixed with 4% paraformaldehyde for 5 min in the dark and washed two times with 5 ml of PBS. The coverslips were mounted on a glass slide (Sigma-Aldrich) containing a drop of Prolong gold anti-fade agent (Molecular Probes, Eugene, OR) and sealed with transparent nail polish.

Visualization of the biofilms was carried out by laser scanning confocal microscopy using an SP8 confocal microscope (Leica Microsystems, Inc., Buffalo Grove, IL), with the laser source settings at 488 nm and 552 nm to visualize P. gingivalis and S. gordonii, respectively. Images were collected using a Z plane scan for a depth of 25 μm using a Z step thickness of 0.7 μm. Background noise was reduced using the software that was supplied with the Leica SP8 microscope. Three-dimensional reconstruction of the Z plane scans and quantification of the red and green channel fluorescence were performed using Volocity 6.3 image analysis software (PerkinElmer, Waltham, MA). The adherence of P. gingivalis to S. gordonii, as determined from the ratio of green channel to red channel fluorescence, was normalized against the PBS control samples. Three independent Z stack images were reconstructed and analyzed from each coverslip, and each treatment was carried out in triplicate. Three independent experiments were performed for each test compound. A pairwise comparison using a *t* test was performed using GraphPad version 8.0.

### Murine *in vivo* model of periodontitis.

The protocol used in this study (protocol 16486) was approved by the Institutional Animal Care and Use Committee at the University of Louisville under federal guidelines for the use and care of laboratory animals. Ten-week-old BALB/c/ByJ specific-pathogen-free mice were obtained from Jackson Laboratory (Bar Harbor, ME) and housed in the University of Louisville Research Resource Center animal facility. During the entire course of the study, mice were fed with Lab Diet 5001 (Purina Mills, LLC, Gray Summit, MO).

Oral infection of the mice was carried out essentially as described by Deap et al. (18). Prior to infection, mice were subjected to a combined antibiotic treatment for 10 days by providing animals with water *ad libitum* containing 800 μg/ml sulfamethoxazole (MP Biomedical, Solon, OH) and 400 μg/ml trimethoprim (Sigma, St. Louis, MO). Four days after the conclusion of the antibiotic treatment, mice were infected by oral gavage with 10^9^ CFU of S. gordonii suspended in 2% carboxymethylcellulose (CMC; MP Biomedical) in sterile PBS using a 2.25-mm feeding needle (Popper and Son, Inc., New Hyde Park, NY). Infections were conducted every other day for a period of 10 days. Subsequently, mice were infected with 10^9^ CFU P. gingivalis suspended in 2% CMC in PBS alone or in 2% CMC in PBS containing the small-molecule inhibitors every other day for 10 days. After the last infection with P. gingivalis, the mice were rested for 47 days and then euthanized by carbon dioxide asphyxiation.

To obtain the mouse skulls, the heads were removed and autoclaved for 15 min to remove flesh and musculature. The defleshed skulls were then immersed in 3% hydrogen peroxide overnight at room temperature to remove any remaining flesh. The skulls were submerged in deionized water to remove residual hydrogen peroxide, soaked in 1% bleach solution for 30 sec, sonicated at 14 V for 1 min, and washed again with deionized water. Samples were brushed using toothpaste, sonicated again in 1% bleach for an additional 30 sec at 14 V, and washed with water. The cleaned skulls were stained by immersion in 1% methylene blue for 15 sec, followed by washing with water until the excess dye was removed.

To measure alveolar bone loss, a dissecting microscope fitted with a video imaging marker measurement system (model VIA-170K; Fryer) was used. Bone loss was determined by measuring the distance between the alveolar bone crest (ABC) and cemento-enamel junction (CEJ) at eight sites on the buccal sides of both left and right maxillary molars. Bone loss was measured in mm for each group of mice, and bone loss data were normalized by subtracting the average bone loss that was observed in sham-infected mice. Data were analyzed using one-way analysis of variance (ANOVA; Graph Pad Prism, La Jolla, CA), and a pairwise parametric analysis of variance using a Bonferroni multiple-comparison posttest was used to determine the statistical difference among the individual mouse groups. A *P* value of ≤0.05 was considered statistically significant.

### Tissue culture.

Human telomerase immortalized gingival keratinocytes (TIGKs) (34) were cultured at 37°C in basal medium from the Dermalife K complete kit with supplements (LifeLine, Frederick, MD) and were incubated for 5 days in an atmosphere of 5% CO_2_ to attain >95% confluence. The mouse macrophage cell line J774A.1 was grown in Dulbecco’s modified Eagle medium (DMEM) (Thermo Fisher Scientific, Waltham, MA) supplemented with 4.5 g/ml glucose, 10% fetal bovine serum (FBS), and 100 U/ml penicillin-streptomycin (Sigma-Aldrich, St. Louis, MO). HL60 (ATCC CCL240) cells were obtained from the American Type Culture Collection and cultured in Iscove’s modified Dulbecco’s medium (Sigma-Aldrich) supplemented with 20% fetal bovine serum (FBS) (Sigma-Aldrich). Both J774A.1 and HL60 cells were incubated at 37°C in an atmosphere of 5% CO_2_ for 4 days to reach >95% confluence.

### Determination of lactate dehydrogenase activity.

The CytoTox 96 nonradioactive cytotoxicity assay (Promega Inc. Madison, WI) was used to determine extracellular lactate dehydrogenase activity (LDH) in TIGK, J774A.1, and HL60 cell cultures treated with the test compounds. Briefly, cells were inoculated in a 96-well microtiter plate at an initial density of 4,000 cells per well and were grown at 37°C in an atmosphere of 5% CO_2_ for 24 h. The spent medium was removed and replaced with fresh medium containing the desired concentration of the compound. After an 18-h incubation, the cell supernatant was collected by centrifugation at 250 × *g* for 4 min, and 50 μl of cell free supernatant was transferred into wells of a fresh 96-well microtiter plate. Subsequently, 50 μl of the LDH substrate was added in each well and incubated at room temperature for 30 min. Reactions were stopped by adding 50 μl of the stop solution according to the manufacturer’s protocol, and the endpoint was measured by determining optical density at 490 nm. Cells treated with culture medium containing 0.1% DMSO or with medium alone served as negative controls, and cells incubated with 15-μl lysis buffer (supplied by manufacturer) for 1 h served as a positive control for lysis. All samples were tested in triplicate.

### Measurement of total cellular ATP.

To determine the metabolic activity of cells, the total cellular ATP level was measured in cell culture samples using the CellTiterGlo reagent (Promega Inc. Madison, WI). Cells were grown as described above in the presence of the desired concentration of compounds. The spent medium was decanted, and cells were washed three times with PBS and subsequently incubated with 100 μl of the CellTiterGlo substrate. To facilitate the reaction, plates were incubated at room temperature for 2 min with shaking and for an additional 10 min without shaking. Total light production was measured using a Victor 3 multilabel plate reader (PerkinElmer).

### Determination of apoptotic activity.

The apoptotic activity of the compounds was determined using the phycoerythrin (PE) annexin V/dead cell apoptosis kit with Sytox green for flow cytometry (Invitrogen, Carlsbad, CA). Cells were grown in 12-well flat-bottom microtiter plates at an initial density 2 × 10^5^ cells in 1.5-ml medium per well for 24 h. Following the incubation period, spent medium was replaced with fresh medium containing the desired concentration of the test compound and incubated for another 18 h. After trypsinization, cells were harvested by centrifuging at 250 × *g* for 4 min, and the cell pellet was suspended in 100 μl of binding buffer supplemented with 1 μl Sytox and 5 μl annexin V, followed by incubation at 37°C for 15 min. Samples were then diluted by adding 400-μl binding buffer, and flow cytometry was performed using a FACScalibur flow cytometer (Becton, Dickinson, Franklin Lakes, NJ), measuring the fluorescence emissions at 530 nm and 575 nm. Cells treated with medium alone or with medium containing 0.1% DMSO served as negative controls, while cells treated with 2 mM hydrogen peroxide at 37°C for 4 h served as a positive control for apoptosis.

### Measurement of hemolytic activity.

The hemolytic activity of compounds was determined using both sheep and human red blood cells. Briefly, 100 μl of 1% sheep or human erythrocytes (BioreclamationIVT, MD) was suspended in 1 ml of sterile PBS containing 5% FBS, and the appropriate concentration of compound was added. The cell suspension was incubated at 37°C for 3 h and centrifuged at 3,500 × *g* for 5 min, and a 200-μl aliquot of the cell-free supernatant was transferred into each well of a 96-well microtiter plate. Hemolytic activity was measured by quantifying hemoglobin release in the supernatant using a Victor 3 multilabel plate reader (PerkinElmer, Waltham, MA) at a wavelength of 538 nm. All samples were analyzed in triplicate. Erythrocytes suspended in PBS with 5% FBS served as a negative control, and erythrocytes that were lysed by suspension in distilled water (dH_2_O) served as a positive control.

### Statistical analysis.

For each of the cytotoxicity assays described above, data were analyzed using a pairwise nonparametric *t* test using GraphPad Prism version 8.0. A *P* value of <0.05 was considered statistically significant.

## Supplementary Material

Supplemental file 1
