# Supplementary material for: Identification of Small-Molecule Inhibitors Targeting Porphyromonas gingivalis Interspecies Adherence and Determination of Their In Vitro and In Vivo Efficacies
Source: Antimicrob Agents Chemother. 2020 Oct 20;64(11):e00884-20. doi: 10.1128/AAC.00884-20 (PMC7577153; doi:10.1128/AAC.00884-20)
Supplement: Supplemental file 1 [file AAC.00884-20-s0001.pdf]

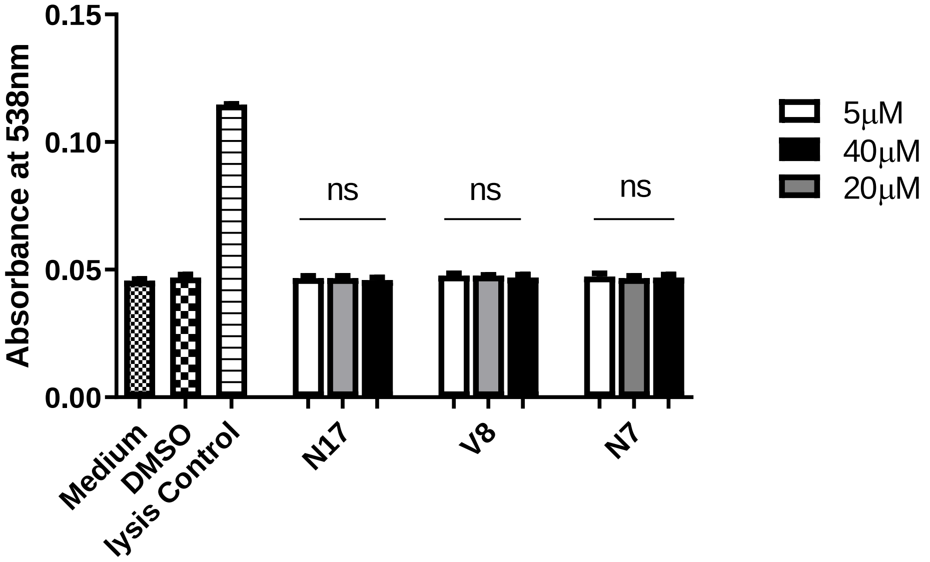

Figure S1. Hemolytic activity of the compounds N7, N17 and V8 against sheep red blood cells after incubation with compounds at concentrations of 5, 20 and 40 μM. ns, not significant.

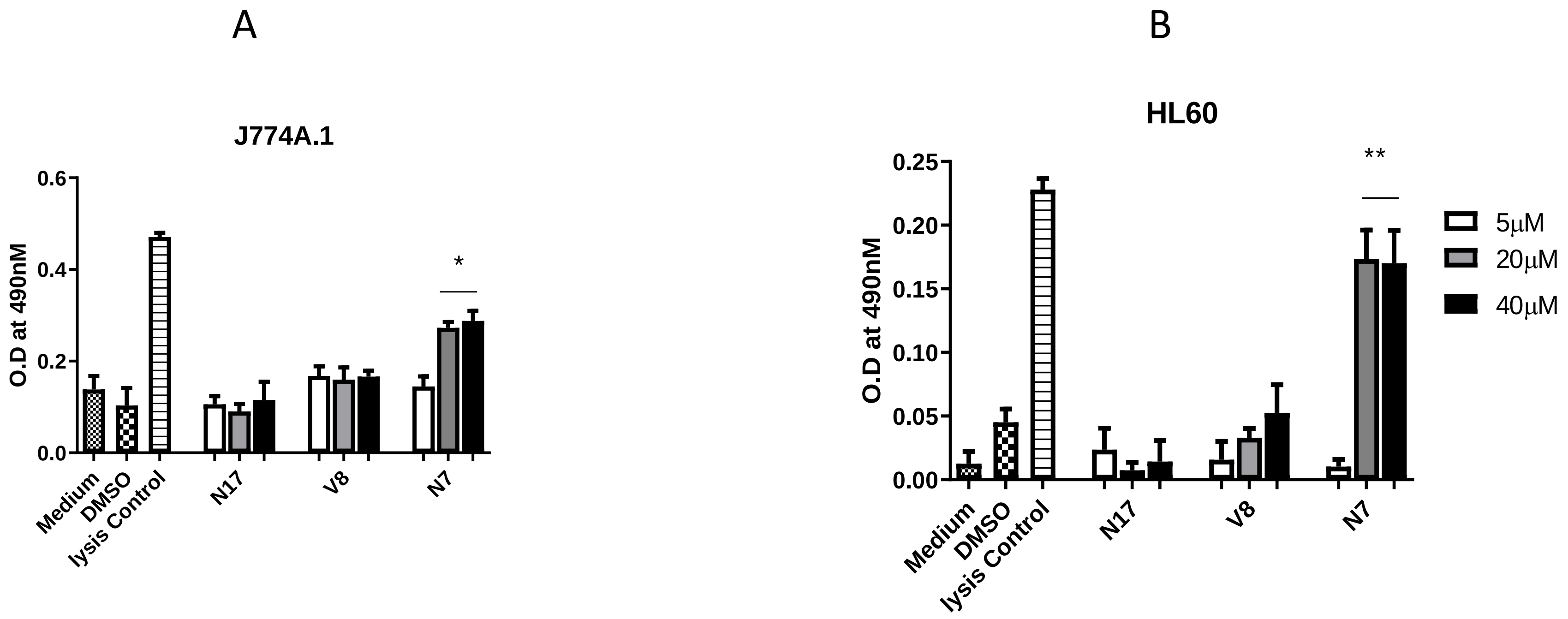

Figure S2. Cytolytic activity of compounds N7, N17 and V8 against J774A.1 (**A**) and HL60 (**B**) cells. LDH activity in cell free medium supernatants was determined after incubation of cells with the compounds for 18 hr at a concentration 5, 20 and 40 μM. *$p<0.05$, **$p<0.01$

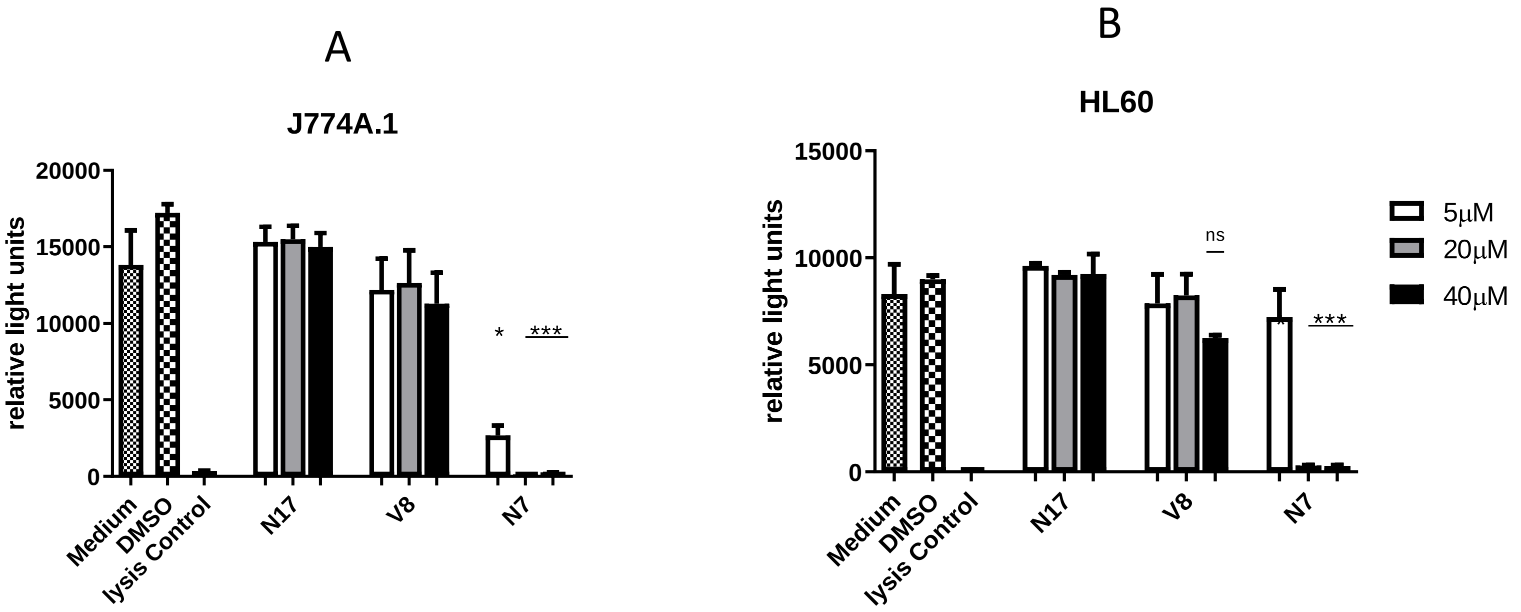

Figure S3. Effect of compounds N7, N17 and V8 on J774A.1 (**A**), and HL60 (**B**) cell viability. Cellular ATP levels were determined after treating cells with the compounds for 18 hr at a concentration 5, 20, 40 µM. Significant differences were determined by comparing experimental samples to the medium only and medium/DMSO controls. *p<0.05, ***p<0.001, ns – not significant.

Table S1. Quantification of early and late apoptotic J774A cells after exposure to compounds N7, N17 or V8.

Treatment	Conc (μM)	Live cells (%)	Early apoptosis (%)	Late apoptosis/necrosis (%)
Medium		89.6	0.1	5.3
Medium/DMSO		89.6	0.9	5.5
Medium/H_2O_2		41.9	1.3	34.7*
N17	5	87.8	1.0	6.1
	20	85.3	1.0	6.9
	40	87.3	1.2	6.6
V8	5	85.1	1.1	8.3
	20	83.4	0.9	8.0
	40	84.3	12.5	9.1
N7	5	83.9	2.5	5.5
	20	33.4	16.9*	46.6*
	40	36.2	24.9*	38.0*

Asterisks indicate a significant increase ($p < 0.001$) in apoptotic cells relative to the medium and medium/DMSO controls.

Table S2. Quantification of early and late apoptotic HL60 cells after exposure to compounds N7, N17 or V8.

Treatment	Conc (μM)	Live cells (%)	Early apoptosis (%)	Late apoptosis/necrosis (%)
Medium		88.9	1.1	6.9
Medium/DMSO		90.0	1.2	6.6
Medium/H_2O_2		52.7	12.3*	31.4*
N17	5	91.3	2.7	4.7
	20	90.3	2.2	6.0
	40	91.6	2.3	6.3
V8	5	91.8	2.9	5.6
	20	91.3	3.7	5.0
	40	88.4	2.4	6.7
N7	5	87.4	3.6	7.8
	20	27.3	20.9*	43.9*
	40	18.9	7.7	70.8*

Asterisks indicate a significant increase ($p < 0.001$) in apoptotic cells relative to the medium and medium/DMSO controls.
